# Identification of myoelectric signals of pregnant rat uterus: new method to detect myometrial contraction

**DOI:** 10.3325/cmj.2017.58.141

**Published:** 2017-04

**Authors:** Kálmán F. Szűcs, György Grosz, Miklós Süle, Anikó Nagy, Zita Tiszai, Reza Samavati, Róbert Gáspár

**Affiliations:** 1 Department of Pharmacodynamics and Biopharmacy, University of Szeged, Hungary; 2 MDE GmbH, Walldorf, Germany; 3 Heim Pál Children's Hospital, Budapest, Hungary

## Abstract

**Aim:**

To develop an electromyography method for pregnant rat uterus *in vivo* and to separate myometrial signals from the gastrointestinal tract signals.

**Methods:**

Pregnant Sprague-Dawley rats (n = 8) were anaesthetized and their stomach, small intestine, and large intestine were removed from the abdomen. A pair of thread electrodes was inserted into the uterus, while a pair of disk electrodes was placed subcutaneously above the myometrium. Additionally, a strain gauge sensor was fixed on the surface of the myometrium and cecum for the parallel detection of mechanical contractions in rats (n = 18) with intact gastrointestinal tract. The filtered electric signals were amplified and recorded by an online computer system and analyzed by fast Fourier transformation. The frequency of the electric activity was characterized by cycle per minute (cpm), the magnitude of the activity was described as power spectrum density maximum (PsD_max_).

**Results:**

The frequency of the pregnant uterine activity was 1-3 cpm, which falls within the same range as that of cecum. Measuring by both electrodes, oxytocin (1 µg/kg) increased and terbutaline (50 µg/kg) decreased the PsD_max_ by 25%-50% (*P* < 0.001) and 25%-40% (*P* < 0.01), respectively. We found a strong positive correlation between the alterations of PsD_max_ values and the strain gauge sensor-detected mechanical contractions (area under curve). The GI specific compounds (neostigmine, atropine) mainly affected the cecal activity, while myometrium specific drugs (oxytocin, terbutaline) influenced the myometrial signals only.

**Conclusion:**

Our method proved to be able to detect the myoelectric activity that reflects the mechanical contraction. The overlapping myometrial and cecal signals are not separable, but they can be distinguished based on the much higher activity and different pharmacological reactivity of the pregnant uterus. Thus, the early signs of contractions can be detected and labor may be predicted in a fast and sensitive way.

Premature labor is the major contributor to perinatal mortality and morbidity, with an average rate of 10%-12% in developing and developed countries and accounts for 75%-85% of all neonatal deaths. Preterm labor is defined as delivery occurring before 37 completed weeks of gestation (1,2). A sensitive method to predict the early signs of term or preterm pregnant uterine contraction would have a great importance in clinical practice. Although a few clinical studies have been published about the application of electromyography in obstetrics (3,4), the characterization of slow wave uterine signals and their comparison to other smooth muscles waves have not been described yet.

The physiology of pregnant uterine contractility is very complex and not yet fully understood. Myometrial contraction is regulated by sex hormones, the autonomic nervous system, ion channels on myometrial cells, and transmitters. Dysregulation of the myometrial contractility can lead to either preterm or slow-to-progress labor (5).

Specialized cells, known as interstitial cells of Cajal (ICCs), have been identified in specific locations within the myenteric plexus. Cajal cells play a key role in several important functions in the gastrointestinal tract, such as generation of electrical slow wave activity, coordination of pacemaker activity, liaison between the enteric nervous system and the GI tract, and mechanosensation to stretch the gastrointestinal muscles (6).

ICCs were immunohistochemically detected using c-kit/CD 117 antibodies in a variety of smooth muscle tissues, including the myometrium. These myometrial CD 117-positive cells could behave as sensors, controlling myometrial contractility, depending on steroid hormone levels. Myometrial ICCs exhibited spontaneous electrical and pacemaker activity and responsibility for the generation of slow myoelectric waves. It has been suspected that CD 117-positive cells are associated with myometrial motility disorders, which may have a role in the pathogenesis of endometriosis and the regulation of labor in the pregnant uterus (7,8).

The electric signals of the smooth muscles have significantly lower frequencies as compared with brain, cardiac or skeletal muscle electric signals. The GI slow-wave signals are well characterized. The stomach, ileum and cecum have different mechanical and myoelectric activities, which are well distinguishable from each other (9). The different myoelectric frequencies of the GI tract segments suggest differences in pacemaker activity and propagation velocity of the ICCs network; however, we have no data about the uterine activity. Therefore, the identification of the signals from the myometrial smooth muscle is a prerequisite for the diagnosis of contractility disorders and the prediction of premature birth.

The aims of our study were to identify and characterize the electric activity of pregnant rat myometrium, separate these signals from the gastrointestinal signals, and develop a method to follow up the changes in myoelectric activity in parallel with the mechanical contractions in vivo.

## Methods

### Ethical approval

The animals were treated in accordance with the European Communities Council Directives (86/609/ECC) and the Hungarian Act for the Protection of Animals in Research (Article 32 of Act XXVIII). All experiments involving animal subjects were carried out with the approval of the Hungarian Ethical Committee for Animal Research (registration number: IV/198/2013).

### Housing handling and mating of the animals

Sprague-Dawley rats (Charles-River Laboratories, Budapest, Hungary) were housed at 22 ± 3°C and a relative humidity of 30%-70%, under a 12 h light/12 h dark cycle. Standard rodent pellet food (Charles-River Laboratories, Budapest, Hungary) and tap water were provided *ad libitum*.

Sexually mature female Sprague-Dawley rats (body mass: 140–160 g, 50–60 days old) were mated in the early morning hours. Copulation was conﬁrmed by the presence of a copulation plug or spermatozoa in the vaginal smear. The day of copulation was considered to be the ﬁrst day of pregnancy.

### Detection of myoelectric activity

Female, full term pregnant (Day 21 and Day 22 of pregnancy) Sprague-Dawley rats were anaesthetized intraperitoneally (i.p.) with a combination of ketamine (36 mg/kg) and xylazine solution (4 mg/kg). The jugular vein was cannulated for later intravenous (i.v.) drug administration.

**Figure 1:**
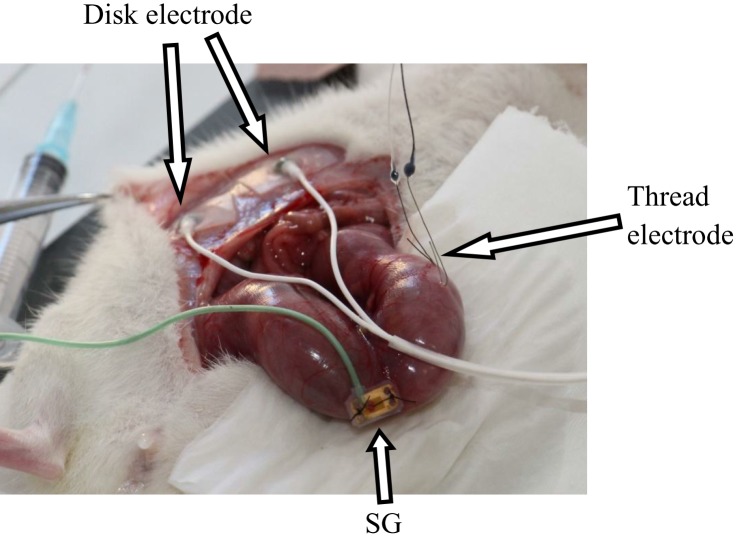
Representative picture of the positioning of the electrodes and strain gauge (SG) for recording the myometrial myoelectric and mechanical signals in a rat with a resected gastrointestinal (GI) tract. The thread electrode pair and the SG were positioned on the uterus, while the disk electrode pair was positioned on the abdomen under deep anesthesia.

In the first group of rats (n = 8), a laparotomy was performed and the total gastrointestinal tract was resected from the abdomen under deep anesthesia. A bipolar thread electrode pair (SEN-15-1; MDE GmbH, Walldorf, Germany) was inserted into the myometrium (the distance between the two electrodes was 8 mm), while a bipolar disk electrode pair (SEN-15-2; MDE GmbH, Walldorf, Germany) was placed subcutaneously above the uterus (the distance between the two electrodes was 20 mm). An implantable strain gauge (SEN-04-FSG2; MDE GmbH, Walldorf, Germany) was sutured onto the surface of the left uterine horn, along the long axis of the muscle fibers, in order to detect the mechanical contractions (Figure 1). So as to cover the incision, the surfaces of the abdominal wall were constricted and the abdominal skin was replaced after the positioning of the sensors. The animals were then placed immediately onto a heatable operating table (EXP-D-TC/MA-02; MDE GmbH, Walldorf, Germany) in order to maintain the body temperature (set to 37°C). The basal activity was detected for 60 minutes. The electric signals were recorded and analyzed by an online computer and amplifier system by the S.P.E.L. Advanced ISOSYS Data Acquisition System (MDE GmbH, Walldorf, Germany). Electromyographic (EMG) signals were ampliﬁed by using a custom-made ampliﬁer. All analogue signals were filtered with a first-order bandpass Bessel-type filter with a frequency of 0-30 cycles per minute (cpm) and were converted to digital signals at a sample rate of 2 Hz.

During the research the animals were under deep anesthesia. When the experiments were completed, we sacrificed the rats with i.v. administered high dose of ketamine (150 mg/kg).

The recorded signals were analyzed by fast Fourier transformation (FFT). The frequency of the electric activity was characterized in cpm, and the magnitude of the activity was described as maximum of power spectrum density (PsD_max_). When more than one peak was found in the spectrum, only the highest peak was considered during the evaluation. The mechanical contractions were evaluated by area under the curve (AUC) analysis of the primary contractility curves. Before the pharmacological studies, both the mechanical (strain gauge) and electric (thread and disk electrodes) signals were recorded for 30 minutes (n = 8).

**Figure 2:**
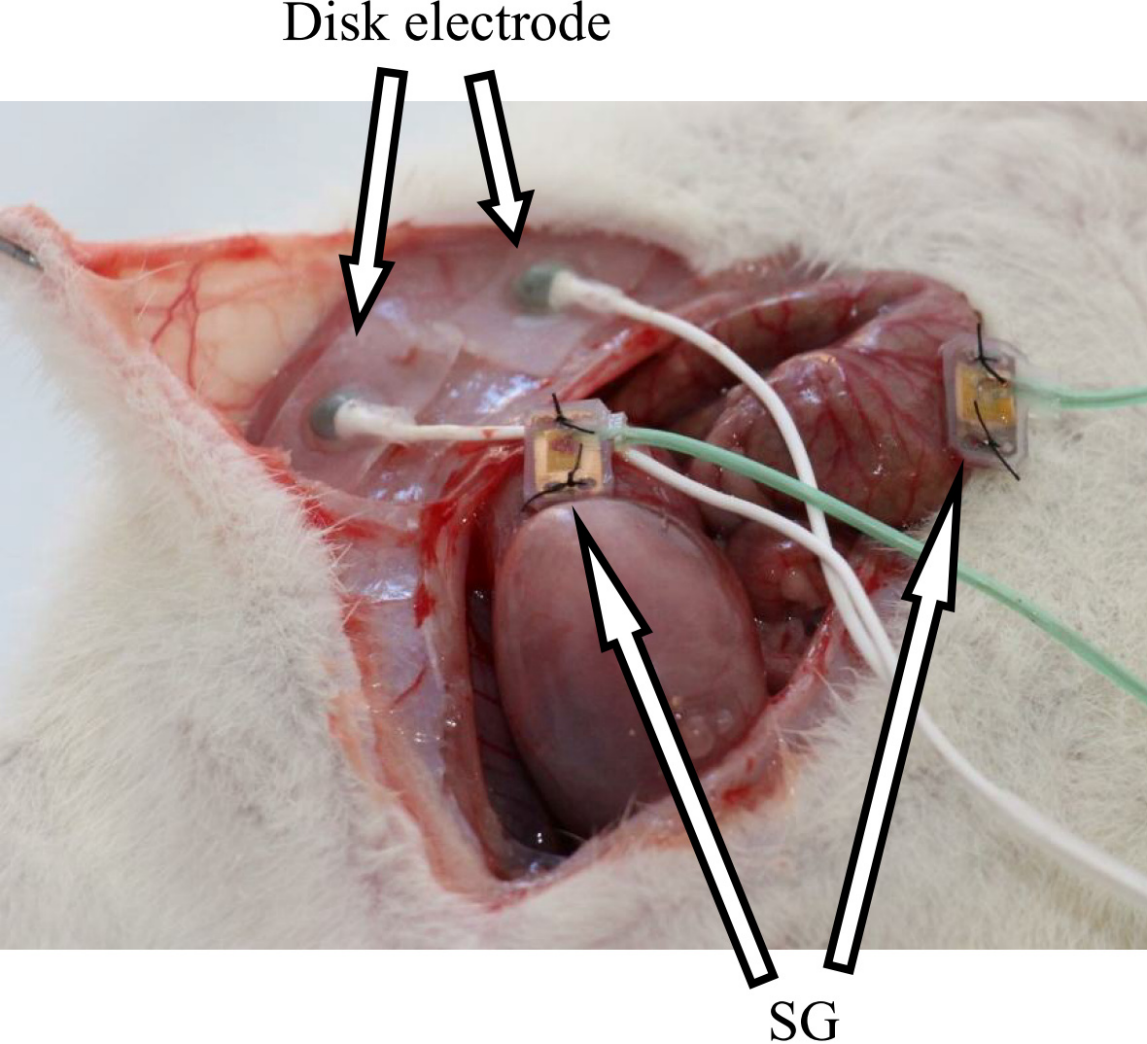
The positioning of the disk electrode and strain gauges (uterus, cecum) for recording of the uterine and gastrointestinal (GI) myoelectric and mechanical signals in a non-GI tract-resected rat under deep anesthesia.

In the second group of anaesthetized rats (n = 18) with intact gastrointestinal tract, a bipolar disk electrode was placed under the abdominal skin, 1 cm right from the midline of the laparotomy, and 2 strain gauges were sutured one by one onto the surface of the uterus and cecum (Figure 2). The abdominal incision surfaces were closed by surgical staples after the placement of the sensors. Both the mechanical (strain gauges) and electric signals (disk electrode) were recorded for 30 minutes before the administration of the investigated drugs. The myoelectric signals were recorded with the above-mentioned equipment. The analysis and filtering (0-3 cpm) of the signals were carried out as it was described in our previous study (9).

### Pharmacological investigations

Two doses of oxytocin (1 μg/kg) were administered after recording the basal activity, 15 minutes apart. After 30 minutes, a dose of terbutaline (50 μg/kg) was injected i.v. to rats with resected gastrointestinal tract (n = 8 on Day 21 of pregnancy) and rats with intact gastrointestinal tract (n = 10 on Day 22 of pregnancy). One dose of neostigmine (20 μg/kg) and one dose of atropine (300 μg/kg) were administered intravenously 30 minutes apart to rats (n = 8) with intact gastrointestinal tract on Day 22 of pregnancy. Following the administration of each drug, the AUC and FFT were evaluated at 30-minute intervals. The effects were expressed as percentages of the spontaneous activity.

### Statistical analysis

The AUC, cpm, and PsD_max_ values were determined and compared using unpaired *t*-test. The *P* values of unpaired *t*-tests indicating statistically significant differences are shown in respective figures. Statistical analyses were performed using the statistical program Prism 5.0. (GraphPad Software, La Jolla, CA, USA), and the level of statistical significance was set at *P* < 0.05.

## Results

**Figure 3:**
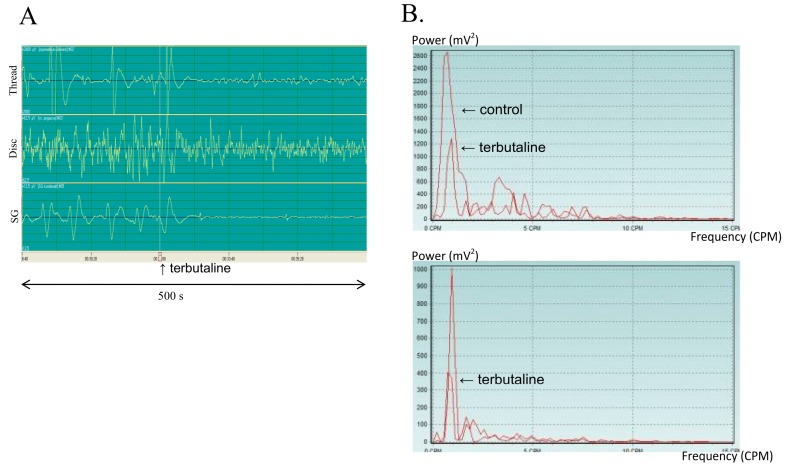
Myoelectric and mechanical signals of the pregnant uterus (**A**), detected with different electrodes and with the strain gauge (SG). Fast Fourier transformation (FFT) analysis reveals the tissue specific spectra of the myometrium (**B**). Each spectrum has the characteristic frequency expressed in cycles per minute (cpm), determined by the highest peak in the spectrum.

Primary EMG curves from rats with resected gastrointestinal tract represent characteristic myoelectric signals recorded by thread or disk electrodes. When the primary EMG curves were transformed by FFT, the resulting spectra had sharp and dominant peaks (PsD_max_) at low cpm values, and terbutaline reduced the PsD_max_ of the spectra (Figure 3).

**Figure 4:**
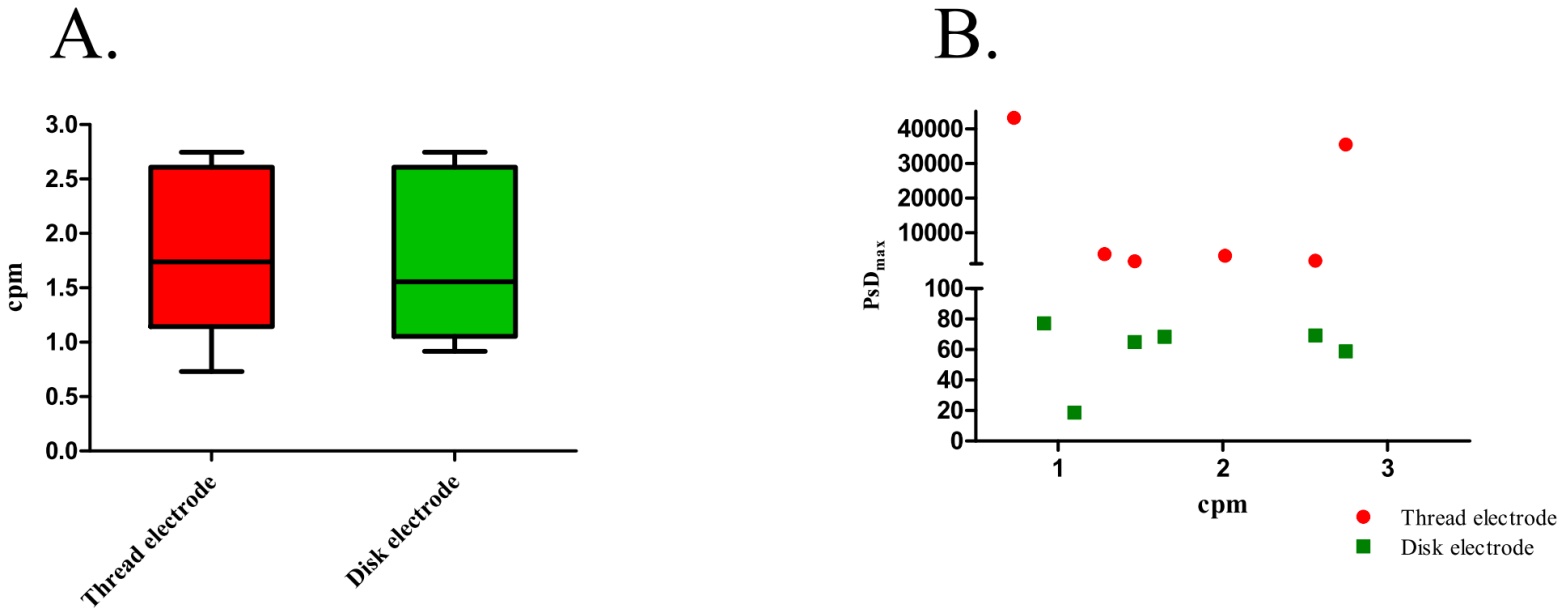
Cycles per minute (cpm) values were measured with thread and disk electrodes (**A**). The intensity of the signals is expressed as the maximum of the power spectrum density (PsD_max_), which corresponds to the highest peak in the Fast Fourier transformation spectrum. The signals of the thread electrodes were usually higher than those of the disk electrodes (**B**).

The characteristic cpm value for the uterus was found between 1-2.5 cpm measured by thread or disk electrodes (Figure 4A). The PsD_max_ representing the maximum intensity of the signals was higher with the thread electrodes in the cpm range 1-2.5 (Figure 4B).

**Figure 5:**
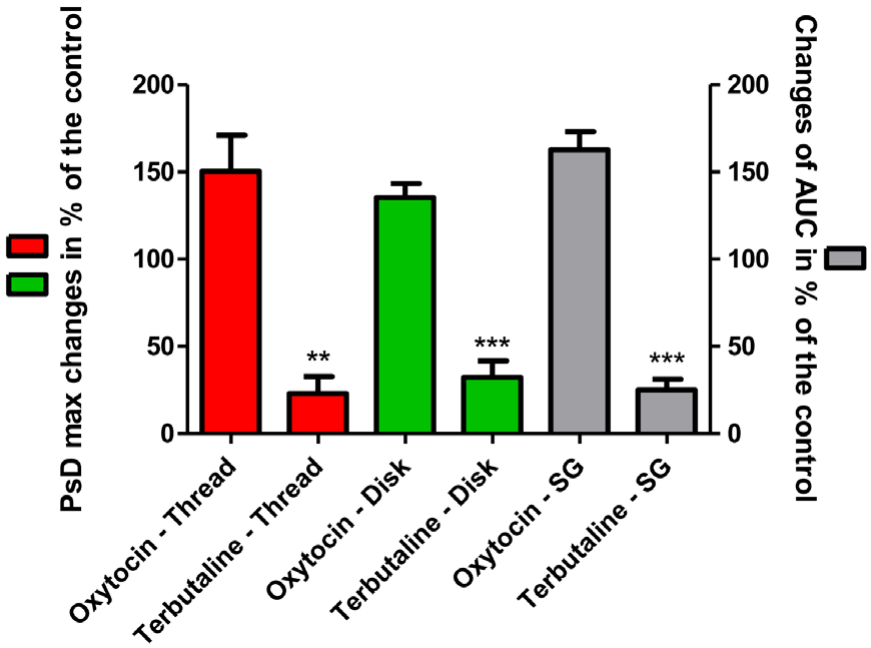
Changes in uterine activity after oxytocin and terbutaline treatments in rats with resected gastrointestinal track. The intensity of the electric signals was expressed as the maximum of the power spectrum density (PsD_max_), while the mechanical contraction was evaluated by area under curve (AUC) analysis. Oxytocin increased, while terbutaline reduced the electric and mechanical activities of the smooth muscles relative to the basic activity (100%) (*P* < 0.05*; *P* < 0.01**; *P* < 0.001***).

**Figure 6:**
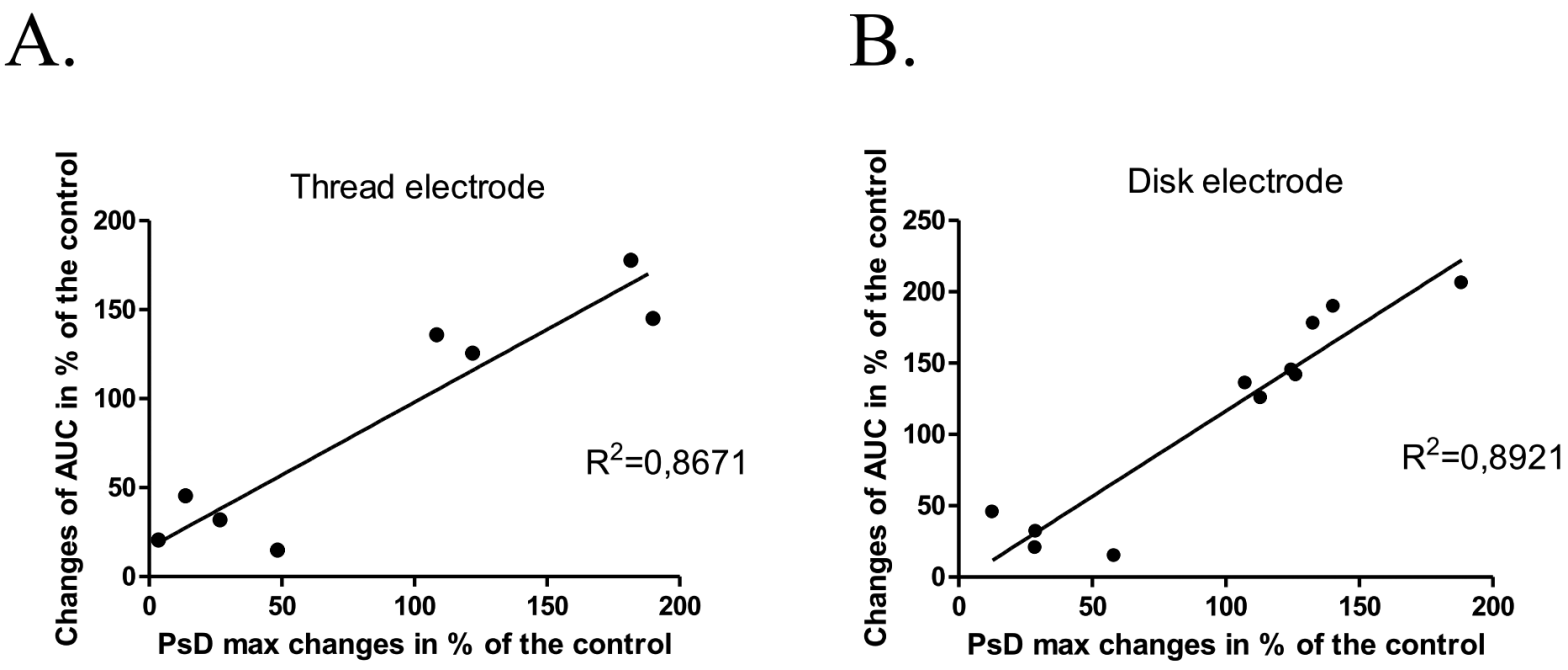
Correlations between myoelectric and mechanical responses induced by oxytocin and terbutaline treatments in GI tract-resected animals. The myoelectric response recorded by thread (**A**) or disk electrode (**B**) is expressed as the maximum of the power spectrum density (PsD_max_), while the mechanical response is expressed as the area under the curve (AUC) of the recorded smooth muscle contractions compared to the basic activity.

In pharmacological experiments oxytocin increased, while terbutaline decreased both the electrical and mechanical signals of the uterus in the rats with resected gastrointestinal tract (Figure 5). The FFT analysis of signals detected by the thread and disk electrodes revealed significant changes in the PsD_max_ values (*P* = 0.0014, and *P* < 0.001; respectively), while the AUC analysis demonstrated similar changes (*P* < 0.001) in the mechanical contractions. The extents of stimulation or inhibition were similar in both mechanical and electrical changes. A statistically significant positive correlation was found between the changes in the PsD_max_ and AUC values measured by thread (Figure 6A) or disk electrode (Figure 6B) in comparison with SG signals (*P* = 0.0008 and *P* < 0.0001, respectively).

**Figure 7:**
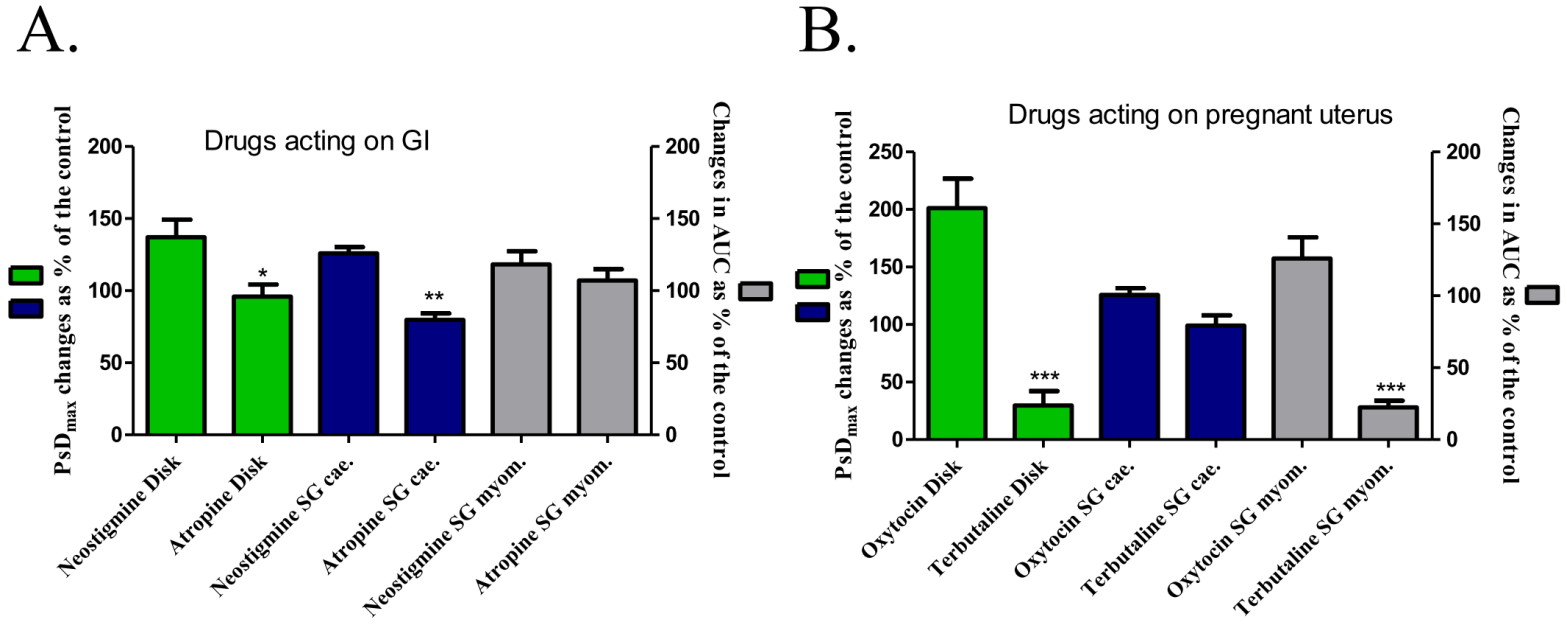
Changes in gastrointestinal (GI) and myometrial smooth muscle activities after neostigmine and atropine (**A**), or oxytocin and terbutaline (**B**) treatments in rats. with intact GI tract. The intensity of the electric signals was expressed as the maximum of the power spectrum density (PsD_max_), while the mechanical contraction was evaluated by area under curve (AUC) analysis (*P* < 0.05*; *P* < 0.01**; *P* < 0.001***).

The myoelectric and contractility responses of cecum and pregnant myometrium have been recorded for different drugs in parallel. We have administered neostigmine and atropine, which mainly act on the GI tract, and oxytocin and terbutaline, which have an effect on the myometrium. Neostigmine and atropine treatment caused significant changes in the myoelectric signal of the cecum (Figure 7A), while oxytocin and terbutaline had an effect on both the electrical and mechanical signals of the uterus only (Figure 7B).

## Discussion

The myoelectric activity of pregnant rat myometrium is measurable from the abdominal surface and well-characterized by cpm value. The intensity of contractions can be expressed and followed by the PsD_max_ values.

The currently used electromyography methods can be distinguished into two main techniques: recording the propagation of the electric waves with a multi-electrode array (10) and investigation of the myoelectric activity with serosal or cutaneous electrode pairs *in vivo* (11).

In the first series of our experiments, the whole gastrointestinal tract was resected for the pure detection of the myoelectric signals from the pregnant uterus. Two pairs of electrodes measured the myoelectric signals: one was inserted into the right horn of the uterus, while the other was laid subcutaneously, above the uterus. The most intensive signals were gained by the organ-inserted electrodes, while the same type of slow wave was detected at the subcutaneous site with a weaker but still well-detectable intensity. This proves that the myometrial myoelectric signals are detectable from the abdominal subcutaneous area, therefore it is possible to get valuable signals of uterine activity from the abdominal surface. The myoelectric activity was measured in parallel with the mechanical contractions by the applied strain gauge. This triple detection along with the gastrointestinal resection and digital filtration provided a clear record of the uterine myoelectric waves in comparison with the contractions.

The frequencies of the electric signals of the smooth muscles, such as pregnant uterus, are significantly lower as compared with brain, cardiac or skeletal muscle electric signals. FFT analysis reveals that the frequency ranges of electroencephalograms, electrocardiograms and skeletal muscle electromyograms are 5-50 Hz, 3-20 Hz, and 10-20 Hz, respectively (12,13). The slow waves of smooth muscle electric activity are usually characterized with a 60-fold lower cpm value of Hz. We found that pregnant uterine smooth muscle frequency is between 1-3 cpm (0.017-0.05 Hz), and therefore the higher frequencies must be cut with the digital filters to filter out the non-uterine myoelectric signals.

The PsD_max_ values usually express the intensity of myoelectric signals. We observed a very good correlation between the changes in mechanical contractile response and the changes in electric PsD_max_ values. This means that the changes in PsD_max_ values recorded even on the abdominal wall perfectly reflect the real changes in the contractions of the uterine smooth muscle. The oxytocin-induced increase and the terbutaline-induced decrease were clearly detectable via the observation of the changes in the PsD_max_ intensity of the myoelectric spectra from pregnant uterus.

In our previous study, we found that the myoelectric spectrum of the cecum can be detected below 3 cpm (9). It means that the myoelectric signals of the pregnant uterus and cecum overlap. Therefore, we performed a second series of experiments on pregnant animals with intact gastrointestinal tract to distinguish between the myoelectric signals from these two types of smooth muscles. The mechanical contractions of both organs were detected by strain gauges applied on the surface of the cecum and pregnant myometrium. It is known that the cecum and pregnant myometrium have different responses to drugs. Cecum is much more sensitive to neostigmine (contraction) and atropine (relaxation), while the pregnant uterus has a very intensive response to oxytocin (contraction) and terbutaline (relaxation) (9,14,15). We found that drugs acting on myometrial activity altered the PsD_max_ with a good correlation to myometrial mechanical contractions, but they did not affect cecal activity. The drugs acting on the cecum elicited similar action on cecal activity without influencing the myometrial response. It means that we can follow the uterine contractions with the evaluation of PsD_max_ values when the uterine activity is predominant. During pregnancy (especially toward the end of pregnancy) the mass of the uterus is significantly increased and its activity is predominant over the cecal contractions. Our method seems to be proper for the detection of the uterine activity in that late-pregnancy period.

The limitation of our study is that we have not yet tested our method in non-anaesthetized rats and, therefore, we have no information about its usefulness in wakeful subjects. Another limitation is that we tested this method only in full-term pregnant rats. Nevertheless, we think that we have successfully developed a new, preclinical method to investigate the pregnant myometrial activity, *in vivo*. Additionally, this method is already suitable for pharmacological investigations for drugs acting on uterine contractions. It also has the potential for the investigation of pathophysiological processes during parturition, even in conscious animals. The method might support the development of new, non-invasive clinical equipment for obstetricians to detect early signs of premature contractions or predict initiation of labor.
